# Comparison of Prevalence and Outcomes of Pediatric Acute Respiratory Distress Syndrome Using Pediatric Acute Lung Injury Consensus Conference Criteria and Berlin Definition

**DOI:** 10.3389/fped.2018.00093

**Published:** 2018-04-09

**Authors:** Samriti Gupta, Jhuma Sankar, Rakesh Lodha, Sushil K. Kabra

**Affiliations:** Department of Pediatrics, All India Institute of Medical Sciences, New Delhi, India

**Keywords:** Pediatric Acute Lung Injury Consensus Conference definition, PALICC criteria, Berlin definition, pediatric acute respiratory distress syndrome, acute respiratory distress syndrome, oxygenation index, oxygen saturation index

## Abstract

**Objectives:**

Our objective was to compare the prevalence and outcomes of pediatric acute respiratory distress syndrome using the Pediatric Acute Lung Injury Consensus Conference (PALICC) criteria and Berlin definitions.

**Methods:**

We screened case records of all children aged 1 month to 17 years of age admitted to the Pediatric Intensive Care Unit (PICU) over a 3-year period (2015–2017) for presence of any respiratory difficulty at admission or during PICU stay. We applied both PALICC and Berlin criteria to these patients. Data collection included definition and outcome related variables. Data were compared between the “PALICC only group” and the “Berlin with or without PALICC” group using Stata 11.

**Results:**

Of a total of 615 admissions, 246 were identified as having respiratory difficulty at admission or during PICU stay. A total of 61 children (prevalence 9.9%; 95% CI: 7.8–12.4) fulfilled the definition of acute respiratory distress syndrome (ARDS) with either of the two criteria. While 60 children (98%) fulfilled PALICC criteria, only 26 children (43%) fulfilled Berlin definition. There was moderate agreement between the two definitions (Kappa: 0.51; 95% CI: 0.40–0.62; observed agreement 85%). Greater proportion of patients had severe ARDS in the “Berlin with or without PALICC group” as compared to the “PALICC only” group (50 vs. 19%). There was no difference between the groups with regard to key clinical outcomes such as duration of ventilation (7 vs. 8 days) or mortality [51.4 vs. 57.7%: RR (95% CI): 0.99 (0.64–1.5)].

**Conclusion:**

In comparison to Berlin definition, the PALICC criteria identified more number of patients with ARDS. Proportion with severe ARDS and complications was greater in the “Berlin with or without PALICC” group as compared to the “PALICC only” group. There were no differences in clinical outcomes between the groups.

## Introduction

Ashbaugh et al. first described acute respiratory distress syndrome (ARDS) as a syndrome of tachypnea, hypoxia, and decreased pulmonary compliance (1). It has been estimated that ARDS accounts for 1–4% of all Pediatric Intensive Care Unit (PICU) admissions, 8–10% of patients requiring mechanical ventilation and estimated mortality of 20–75% despite advances in the management (2–4). Due to high mortality rates, ARDS remains the ultimate challenge in PICU in terms of management and outcome.

The first consensus definition for ARDS was given by American-European Consensus Conference with well-defined criteria for ARDS and ALI (5). Thereafter, in 2012, Berlin definition of ARDS was proposed with few modifications (6). However, both were applicable more to the adult population and despite the different epidemiology and outcomes of pediatric acute respiratory distress syndrome (PARDS), these were applied to children without modification (7). To address this issue, the Pediatric Acute Lung Injury Consensus Conference (PALICC) was convened to propose specific definitions for PARDS in 2014. Notable differences in the PALICC definition are use of oxygenation index (OI) instead of PaO_2_/FiO_2_, option of using SpO_2_-based indices, and less restrictive radiographic criteria. Also, it includes chronic lung disease (CLD) and cardiac conditions [congenital heart disease (CHD)] which contribute to a significant number of patients with ARDS which were previously excluded (8).

While the new definitions have been proposed to address the limitations of using definitions such as AECC or Berlin originally proposed for only adult patients with ARDS, there is a need to test these new definitions before applying them globally in units across the world. For example, if the definitions are too sensitive, patients without ARDS will be wrongly labeled as ARDS and this will increase the resource utilization in a particular unit. While in a resource replete setting this may not impinge on the resources, it will affect the already limited resources in a resource restricted setting. On the other hand, if the definitions are too specific, only the sickest patients with ARDS may be identified and this may lead to delayed interventions and worse outcomes in those with mild to moderate ARDS at admission. Unfortunately, there is a paucity of data evaluating the applicability of the definitions to pediatric population from different settings. There is only one study from a resource replete setting till date (9) and this may not suffice to generalize the applicability of these definitions to all others. The patient profile, disease severity, disease conditions, and time of presentation would affect the prevalence and outcomes of ARDS in different settings. There is a need to evaluate the utility of these definitions in resource restricted settings as well in order to identify the mild and early ARDS cases so that the outcomes could be improved by effective utilization of the limited resources available. Our aim was to compare the PALICC definitions with the Berlin definitions to estimate the prevalence and outcome of PARDS in our setting which is representative of similar settings from low-middle income countries (LMIC).

## Materials and Methods

### Study Setting and Participants

This was a retrospective chart review over a period of 3 years, from 2015 (January) to 2017 (December) in children aged 1 month to 17 years of age admitted to the PICU of a tertiary care teaching hospital. Case records of all patients admitted to the PICU during the study period were screened to identify patients with respiratory difficulty. Children having PICU stay of less than 6 h were excluded. The study was approved by the Institute Ethics Committee.

### Objectives and Outcome Measures

Our objective was to compare the prevalence and outcomes of PARDS using the PALICC criteria (8) and Berlin definition (6).

### Data Collection

We screened records of all eligible patients (patients with respiratory difficulty) and applied both PALICC criteria and Berlin definitions for diagnosis of ARDS. Children fulfilling either of the two criteria were enrolled in the study. Clinical and radiographic data were extracted from the medical records for up to 7 days or until death or extubation, whichever occurred earlier. Pediatric Index of Mortality-3 (PIM-3) score variables at admission, arterial blood gas (ABG) measurements, oxygenation-related variables, ventilator modes, and settings were recorded on day of diagnosis, at 24 h, day 3, and day 7. All patients suspected to have ARDS (those who fulfilled the oxygenation criteria) are subjected to echocardiography to confirm non-cardiogenic origin of pulmonary edema as per our unit protocols. We collected data on adjuvant therapies used in ARDS, including steroids, sildenafil, inhaled nitric oxide, prone position, and use of other support modalities (e.g., inotropes, continuous renal replacement therapy, and extracorporeal membrane oxygenation). Sequential pediatric logistic organ dysfunction (PELOD) score was used for assessing progressive organ dysfunction (10).

We used lung protective ventilator strategies in pressure control or pressure regulated volume control mode with pPeak <35 cm H_2_O, pPlat <30 cm H_2_O, high PEEP (please provide a number), FiO_2_ to keep saturations >88% and permissive hypercarbia prior to publication of PALICC guidelines. Patients on maximal settings were switched to high-frequency oscillatory ventilation (HFOV) as per our protocol (11–13). After publication of the PALICC guidelines, our protocol was modified with upper limit of Pplat of 28 cm of H_2_O and PEEP of upto 15 cm of H_2_O or higher in severe ARDS provided patient was hemodynamically stable. We used HFOV as rescue therapy if Pplat requirement was more than 28 cm of H_2_O after these guidelines.

### Definitions

We defined respiratory difficulty for non-intubated patients as respiratory rate above upper limit of normal as per age, chest indrawing with or without hypoxemia (SpO_2_ at room air <94%) or requirement of any respiratory support (14) and for intubated patients as pressure requirements or oxygen supplementation to keep SpO_2_ >88% but with OI <4 or oxygen saturation index (OSI) <5 (8). The variables used in PALICC criteria were—duration of onset of acute illness to ARDS of less than 7 days, origin of pulmonary edema (cardiogenic or non-cardiogenic), any new infiltrate on Chest X- ray (unilateral or bilateral) and oxygenation defect based on PaO_2_/FiO_2_ (P/F) or SaO_2_/FiO_2_ (S/F) ratio if on non-invasive mechanical ventilation, and OI or OSI if on conventional mechanical ventilation (CMV) (8). OI was calculated using the formula: [mean airway pressure (MAP) × FiO_2_]/PaO_2_ and oxygen saturation index (OSI) using the formula: [MAP × FiO_2_]/SpO_2_. The criteria used in Berlin definition are similar except for bilateral infiltrates on chest X-ray and oxygenation defect based on P/F ratio alone (6). The other differences are—children with CHD and CLD fulfilling the oxygenation criteria are not included in the Berlin definition but included in the PALICC criteria. Patients with invasive mechanical ventilation were stratified into mild, moderate and severe ARDS on the basis of P/F ratio in “Berlin with or without PALICC” group and by OI/OSI in “PALICC only” group.

Sepsis was defined as Systemic Inflammatory Response Syndrome in the presence of or as a result of suspected or proven infection (15). Pneumonia was defined as lower respiratory tract infection associated with fever, respiratory symptoms, and evidence of parenchymal involvement by physical examination or presence of infiltrates on chest X-ray (16). Steroid exposure was defined as any use of systemic corticosteroids. PELOD score was used to assess organ dysfunction (10). Early use of HFOV in ARDS was defined as elective HFOV and use of HFOV after failure/complications of conventional modes was defined as rescue HFOV (17).

### Statistical Analysis

Data were entered into Microsoft Excel 2016 and analyzed using Stata 11.2 (Stata Corp, College Station, TX, USA). Categorical data were presented as number and percentages and continuous data as mean/medians and SD/interquartile ranges (IQR). Chi square test, Ranksum and *t*-test were applied for categorical and continuous variables, respectively. All statistical tests were two-tailed and the significance level was taken as *p* < 0.05. For evaluating the agreement between the two definitions, we used kappa statistics.

## Results

**Figure 1:**
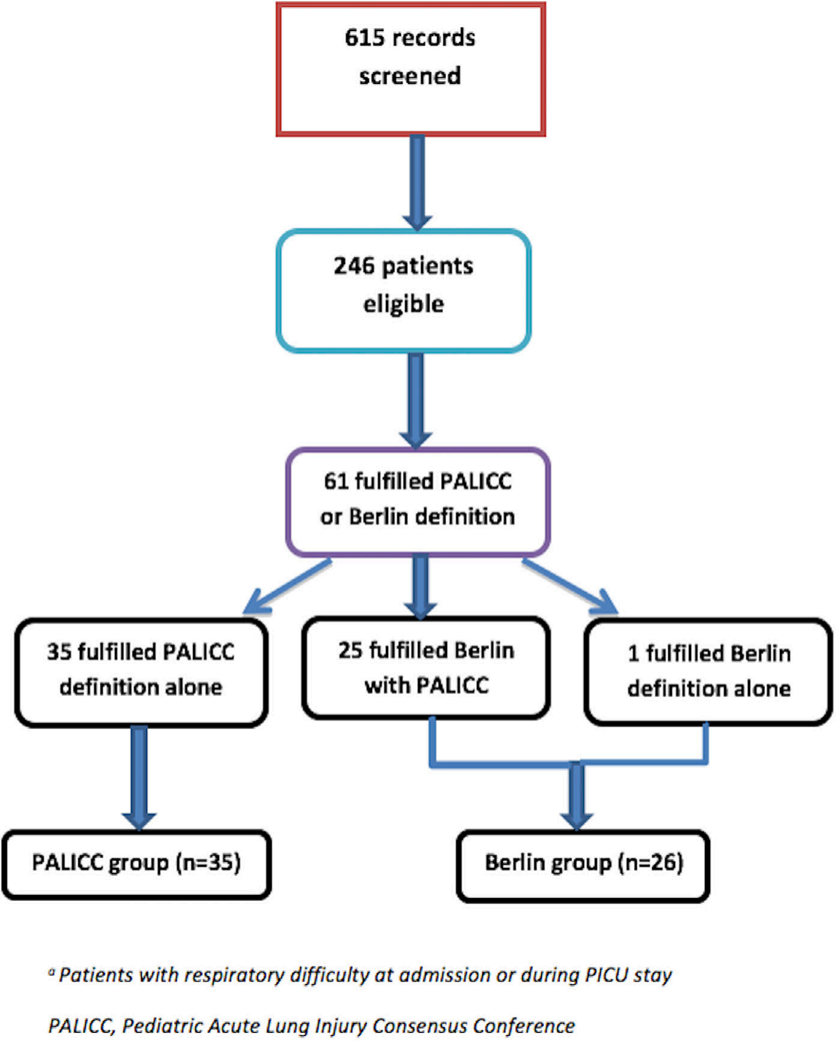
Study flow. *Patients with respiratory difficulty at admission or during Pediatric Intensive Care Unit (PICU) stay.

A total of 615 children were admitted to the PICU during the study period. Of these, 246 were identified as having some respiratory difficulty at admission or during PICU stay. Of these, 61 children fulfilled either Berlin or PALICC criteria. While 60 children (98%) fulfilled PALICC criteria, only 26 children (43%) fulfilled Berlin definition. The patients were divided into “*PALICC only*” group (*n* = 35) and the “*Berlin with or without PALICC*” group (*n* = 26) for comparison of baseline characteristics and various outcomes (Figure 1).

### Primary Outcomes

The prevalence of ARDS in our PICU was 9.9% (95% CI: 7.8–12.4) with either criteria. Prevalence of ARDS with PALICC criteria was 9.75% (4.1, 7.8) and with Berlin definition was 4.2% (2.9, 6.1) (*p* < 0.0001). While 60 children (98%) fulfilled PALICC criteria of the 246 children with respiratory difficulty, only 26 children (43%) fulfilled Berlin definition. There was moderate agreement between the two definitions (Kappa: 0.51; 95% CI: 0.40–0.62; observed agreement 85%).

The baseline characteristics of patients in both groups are shown in Table 1. Compared to “Berlin with or without PALICC” group, “PALICC only” group had lower median (IQR) age [10 (3, 60) months vs. 84 (55, 127) months] (*p* = 0.001). Thirty eight percent (*n* = 23) of the study population had unilateral infiltrates and were included in the “PALICC only” group. The proportion of patients with CHD and CLD fulfilling the standard PALICC criteria were 16% (10) and 7% (4), respectively, in the entire cohort. All patients had non-cardiogenic origin of edema and were on ventilatory support at the time of diagnosis.

**Table 1:** Baseline characteristics of patients in the “PALICC only” and the “Berlin with or without PALICC” definition groups.

Variable	“PALICC only” group (*N* = 35)	“Berlin with or without PALICC” group (*N* = 26)	*p*-Value
• Age in months [median, interquartile range (IQR)]	10 (3, 60)	84 (55, 127)	0.001

• Gender (Males, *N*, %)	19 (54.3)	17 (65.4)	0.43

• Interval between onset of acute illness and diagnosis of acute respiratory distress syndrome (ARDS; median, IQR)	4 (2, 5)	6 (4, 7)	0.005

• Acute illness (pneumonia vs. others) (*N*, %)	28 (80)	16 (61.5)	0.15

**Primary ARDS**			
○ Pneumonia	28 (80)	15 (57.7)	0.03
○ Aspiration	2 (5.7)	1 (3.8)	0.79
**Secondary ARDS**			
Sepsis	6 (17)	9 (34.6)	0.13

• Underlying chronic lung disease (*N*, %)	4 (11.4)	0	0.12

• Underlying heart disease (*N*, %)	10 (28.6)	1 (3.8)	0.04

Respiratory variables			
○ SpO_2_ at diagnosis (Mean, SD)	94 (6.4)	90.0 (11.2)	0.08
○ PaO_2_ at diagnosis (Mean, SD)	82 (67, 104)	90 (56, 134)	0.79
○ FiO_2_ at diagnosis (Mean, SD)	72.7 (23.3)	83.7 (21.9)	0.07
○ Mean airway pressure at diagnosis (Mean, SD)	10.9 (3.9)	13.8 (5.8)	0.02
○ OI^a^ at diagnosis (Median, IQR)	7.9 (4.1, 13.8)	9.6 (6.3, 24.8)	0.11
○ OSI^b^ at diagnosis (Median, IQR)	8.4 (5.2, 11.2)	10.5 (7, 18.7)	0.07
○ P/F^c^ ratio at diagnosis (Median, IQR)	144 (81, 205)	104 (67, 180)	0.20
○ S/F^d^ ratio at diagnosis (Mean, SD)	142.9 (51.5)	118.6 (45.7)	0.06

• Pediatric Index of Mortality-3 probability (Median, IQR)	32 (18, 61)	31 (17, 91)	0.64

• Pediatric logistic organ dysfunction (PELOD) score at 24 hours (Median, IQR)	13 (10, 22)	11.5 (0, 22)	0.23

• Mode of ventilation at diagnosis			
○ Non-invasive (*N*, %)	4 (11.4)	5 (19.2)	
○ Invasive (*N*, %)	31 (88.6)	21 (80.7)	0.39
PSIMV^e^ vs others (*N*, %)	33 (94.2)	20 (76.9)	0.04

• CXR (bilateral infiltrates) (*N*, %)	12 (23.3)	26 (100)	<0.001

Severity of ARDS (*N*, %)	(*n* = 32)	(*n* = 26)	
○ Mild	16 (50)	5 (19)	0.52
○ Moderate	10 (31)	8 (31)	0.51
Severe	6 (19)	13 (50)	0.67

• Multi-organ dysfunction at diagnosis (*N*, %)	26 (74.2)	24 (92.3)	0.09

• Proportion with shock at diagnosis (*N*, %)	23 (65.7)	18 (69.2)	0.89
• Duration of shock (hrs) (Median, IQR)	33 (11, 108)	36 (10.5, 78)	0.86

• Proportion of patients with sepsis (*N*, %)	34 (97.1)	26 (100)	1.00
• Culture positivity (*N*, %)	14 (41.2)	11 (42.3)	1.00
• Nosocomial sepsis (*N*, %)	11 (31.4)	5 (19.2)	0.2
○ Ventilator associated pneumonia (*N*, %)	3 (9)	2 (8)	
○ Catheter related Blood stream infections (*N*, %)	6 (17)	2 (8)	
○ Urinary tract Infections (*N*, %)	2 (6)	1 (4)	

*^a^OI, oxygenation index; ^b^OSI, oxygen saturation index; ^c^P/F, PaO_2_/FiO_2_ratio; ^d^S/F, SpO_2_/FiO_2_ ratio; ^e^P-SIMV, pressure controlled synchronized intermittent mandatory ventilation*.

The most common admitting diagnosis was pneumonia. According to precipitating causes, 75% of cases in the study population had primary ARDS (due to pneumonia or aspiration) and the remaining 25% had ARDS secondary to sepsis (Table 1). Greater proportion of children in the “PALICC only” group had pneumonia and aspiration (86 vs. 62% in the other group) as the cause for ARDS. Sepsis was the precipitating cause in most cases in the “Berlin with or without PALICC” group (35 vs. 17% in the “PALICC only” group).

Other baseline characteristics including PIM-3 probability of death and precipitating events were similar in both groups (Table 1). Max FiO_2_ and minimum PaO_2_ was lower in the “PALICC only” group while lowest SpO_2_ and maximum MAP were comparable (Table 1). Among oxygenation indices, OI (7.9 vs. 9.6) and OSI (8.4 vs. 10.5) were lower, while the P/F (144 vs. 104) and S/F ratios (142.9 vs. 118.6) were higher in the “PALICC only” group as compared to the “Berlin with or without PALICC group,” respectively. The proportion of patients with severe ARDS measured by oxygenation indices—P/F ratio and OI/OSI was higher in “Berlin with or without PALICC group” as compared to “PALICC only” group but the difference was not statistically significant (Table 1). Majority of children in both groups were on invasive mechanical ventilation at the time of diagnosis of ARDS (89 and 81% in “PALICC only” and “Berlin with or without PALICC” groups, respectively) with pressure controlled synchronized intermittent mandatory ventilation (P-SIMV) mode being the commonest mode. The proportion of patients with admission diagnosis of sepsis were similar in both groups (97.1 and 100% in “PALICC only” and “Berlin with or without PALICC” groups, respectively), while those with nosocomial sepsis was higher in “PALICC only” group (32.4 vs 19%) with majority having catheter-associated bloodstream infections in both groups (55 and 40%, respectively) (Table 1).

### Secondary Outcomes

On comparing the key clinical outcomes, proportion of patients requiring HFOV, new air leaks and need for inotropes was higher in the “Berlin with or without PALICC” group; the differences were not statistically significant (Table 2). Proportion of patients with failed non-invasive ventilation [5 (100%) vs 3 (75%), *p* = 0.24], those requiring HFO ventilation [9 (29%) vs 4 (13.3%), *p* = 0.02] and those having air leaks [7 (22.5%) vs 2 (6.7%), *p* = 0.02] was higher in “Berlin with or without PALICC” group as compared to “PALICC only” group. Other outcome variables such as median duration of mechanical ventilation, ICU, and hospital stay were similar in both groups. The overall mortality was similar in both groups (51.4 and 57.7%, respectively) while ARDS-related mortality was higher in “Berlin with or without PALICC” group as compared to “PALICC only” group [7 (46.6%) vs. 3 (16.7%), *p* = 0.1] (Table 2). The non-ARDS related mortality was predominantly due to refractory septic shock and was higher in “PALICC only” group as compared to “Berlin with or without PALICC” group [15 (77.8%) vs 7 (53.3%)]. Of this, nosocomial sepsis contributed to nearly 44% (8/18) of mortality in “PALICC only” group and to 25% (4/15) in the “Berlin with or without PALICC” group (Table 2).

**Table 2:** Comparison of outcomes between the “PALICC only” and the “Berlin with or without PALICC” definition groups.

Variable	“PALICC only” group (*N* = 35)	“Berlin with or without PALICC” group (*N* = 26)	*p*-Value
Proportion of patients with failed non- invasive ventilation at diagnosis (*N*, %)	3 (8.5%)	5 (19%)	0.236

Duration of mechanical ventilation (hrs.) (Median, IQR)			
– Total duration	7 (3.8, 14)	8 (4, 12)	0.816
– Duration of ventilation for acute respiratory distress syndrome (ARDS)	6 (3.8, 10)	7 (2, 12)	0.980

– Proportion of patients requiring HFOV^a^ (*N*, %)	4 (13.3)	9 (29)	0.023
– Duration of HFOV^a^ (hrs) (Median, IQR)	2.5 (0.3, 2.5)	3 (0.4, 7)	0.547

Air leaks (*N*, %)	2 (6.7)	7 (22.5)	0.021

– Need for Vasoactive therapy (*N*, %)	28 (80)	25 (96.1)	0.065
– Duration of Vasoactive therapy (hrs.) (Median, IQR)	52 (24, 96)	18 (7, 72)	0.203

Need for dialysis (*N*, %)	10 (28.6)	5 (19.2)	0.548

– Use of steroids (*N*, %)	22 (62.8)	21 (80.8)	0.171
– Duration of steroids use (days) (Median, IQR)	4.4 (2, 8)	4.8 (1.3, 12)	0.772

Duration of ICU^b^ stay (days) (Median, IQR)	7 (5, 14)	8 (4, 13)	0.775

Duration of hospital stay (days) (Median, IQR)	13 (7, 18)	12 (5, 23)	0.898

PELOD Score (Median, IQR)			
– Day 2	20 (11, 22)	11.5 (3, 22)	0.245
– Day 5	11.5 (1, 21)	11 (2, 11)	0.327

– Mortality (*N*, %)	18 (51.4)	15 (58)	0.966
– Mortality due to ARDS (*N*, %)	3 (9)	7 (27)	0.104
– Mortality due to nosocomial sepsis (*N*, %)	8 (23)	4 (15)	0.49

*^a^HFOV, high-frequency oscillatory ventilation; ^b^ICU, intensive care unit*.

### Severity of ARDS and Clinical Course

On stratifying based on severity of ARDS as per Berlin criteria, the proportion of patients with mild, moderate, and severe ARDS were 19% (5), 31% (8), and 50% (13), respectively. As per OI/OSI criteria (PALICC criteria), 50% (16) had mild, 31% (10) had moderate, and 19% (6) had severe ARDS (Table S1 in Supplementary Material). The proportion of patients with nosocomial sepsis in mild ARDS category in the “PALICC only” group was higher than severe ARDS in the same group (*n* = 8/16, 50% vs. *n* = 2/6, 33%). In the “Berlin with or without PALICC” group, the proportion with nosocomial sepsis was similar in the mild and severe categories (20 vs. 23%, respectively). Children with *severe ARDS* in both “PALICC only” group and “Berlin with or without PALICC” groups had higher need for vasoactive therapy (100%) and steroids (100%). HFOV requirement was 58 and 33% in the *severe ARDS* cases in the “Berlin with or without PALICC” group and “PALICC only” groups, respectively. The overall mortality was also higher in children with *severe ARDS* in both the groups (69 vs 100% in the “PALICC only” group and “Berlin with or without PALICC” groups, respectively). Mortality attributed to ARDS was higher in “severe ARDS category” in the “Berlin with or without PALICC” group (46%, *n* = 6/13) while it was higher in moderate ARDS category in “PALICC only” group (20%, *n* = 2/10). Mortality due to nosocomial sepsis in “mild ARDS category” in “PALICC only” group was 31% (*n* = 5/16) as compared to 20% (*n* = 1/5) in the “mild ARDS category” of “Berlin with or without PALICC group.” There were no differences in other clinical outcomes, i.e., duration of mechanical ventilation and HFOV, steroid use and PICU stay between the mild, moderate and *severe ARDS* cases in both groups (Table S1 in Supplementary Material).

## Discussion

The prevalence of ARDS in our study was 9.9% (95% CI: 7.8–12.4). Prevalence was higher—9.75% (4.1, 7.8) with PALICC criteria (35 patients) as compared to the Berlin definition—4.2% (2.9, 6.1) (26 patients) with moderate agreement between the two (Kappa: 0.51; 95% CI: 0.40–0.62; observed agreement 85%). The probable reasons/hypothesis for the difference in prevalence observed by using the PALICC and Berlin criteria could be—(1) Use of pulse oximetry-based criteria helped in identification of more patients with ARDS which were ignored by Berlins’ criteria due to lack of ABG reports. For example, in our cohort in about 5% of patients, ABG values were not available. In these patients, we used the pulse oximetry based criteria (OSI and or S/F ratio) to diagnose ARDS and, therefore, they could be included in the “PALICC only” group, (2) the chest X- ray criteria of PALICC definition involves any new parenchymal opacity, whether unilateral or bilateral. We observed more than one-third of the study population fulfilling the standard oxygenation criteria for PALICC to have only unilateral findings on chest X-ray. This group of patients comprised almost 67% of the total patients diagnosed by PALICC criteria alone which were missed by the Berlin definition which mandates presence of bilateral symmetrical opacities on chest X-Ray, (3) including children with CLD and CHD in the new PALICC criteria also increased the proportion of patients identified to have ARDS. One quarter of the children had CHD and CLD and could not be included in the Berlin definition.

Many of the published reports of ARDS in PICU have reported the prevalence of ARDS to vary from 0.7 to 4.2% using either AECC or Berlin definitions (2–4, 18). In a previous study from our center, the reported prevalence rate was 20.1/1000 admissions (19). Recently, Parvathaneni et al. reported prevalence of ARDS to be 5.8% by PALICC criteria in their study (9). In their study, the authors had similarly compared the prevalence of ARDS by PALICC criteria and Berlin definitions. Of the 4,764 admissions, the proportion fulfilling PALICC criteria were 5.8% (*n* = 278) while those fulfilling Berlin definition were 3% (*n* = 143) which was about 50% of those fulfilling PALICC criteria. Similar to this study, we observed that only about 40% of patients fulfilling the PALICC criteria fulfilled Berlin definitions. With these observations from previous studies and ours it would be safe to suggest that the Berlin definitions should no longer be used in children for clinical or research purposes and only the PALICC criteria may be used in resource replete or LMIC settings.

While PALICC criteria only were fulfilled by 35 patients, Berlin definition alone was fulfilled by only one patient. This patient had P/F ratio of 293 (PF ratio <300 for fulfilling Berlin’s definition) but OI of 2.9 (OI of >4 for PALICC criteria). Among the other criteria for ARDS, this child had bilateral infiltrates on chest X-ray and was admitted with a diagnosis of pneumonia. He required invasive CMV at diagnosis with initial PEEP of 5 and MAP of 8 cm of H_2_O. The duration of ventilation and PICU stay were 18 and 24 days, respectively.

According to the precipitating causes, ARDS is divided into primary and secondary ARDS. In our study, majority had primary ARDS while only one quarter had secondary causes for ARDS. Pneumonia was the most common precipitating cause of primary ARDS while sepsis was the most common cause of secondary ARDS similar to previous reports (2, 19–21).

A significant difference between the PALICC criteria and Berlin definitions was the use of OI or OSI and discontinuation of the PaO_2_/FiO_2_ ratio to grade the severity of ARDS. By adding MAP into the calculation, the effect of positive pressure on oxygenation was included more objectively (8, 19). More number of patients required HFOV and died in the severe groups by both definitions. The difference, however, was not statistically significant. This could probably be explained by the small numbers in the mild, moderate, and severe groups in both “PALICC only” group and the “Berlin with or without PALICC” groups.

The overall mortality reported in our study was 57.3% which is lower than our previous report of 2001 (75%) (19). The mortality rates reported in various pediatric studies from resource replete and restricted settings has ranged from 22.7 to 63% (2, 9, 18). The mortality in “Berlin with or without PALICC” group was 57.7% and in the “PALICC only” group was 51.4% which was much higher than the study by Parvathaneni et al. who reported a mortality of 32.2 and 22.7% in the “Berlin with or without PALICC” group and the “PALICC only” groups, respectively (9).

The higher mortality observed in our study could be attributed to patients presenting with more severe illness to our unit and differences in resource allocation between our unit and those from resource replete settings. The proportion of patients with ARDS related mortality was higher in the “Berlin with or without PALICC” group as compared to “PALICC only” group (46.6 vs 16.7%), which could probably be due to more severe ARDS patients in “Berlin with or without PALICC” group. Almost half of the patients in the “Berlin with or without PALICC” group had severe ARDS as compared to only 20% in the “PALICC only” group.

The non-ARDS-related mortality was higher in the “PALICC only” group as compared to “Berlin with or without PALICC” group (49 vs 31%) which was predominantly attributed to refractory septic shock and multi-organ dysfunction. One patient had cardiogenic shock as the cause of mortality. A majority of these patients had mild ARDS which initially improved, but subsequently these patients developed multi-organ dysfunction secondary to nosocomial sepsis and majority succumbed to their illness. The patients in the “PALICC only” group had lower ARDS related mortality probably owing to early identification and intervention. This could have improved the outcomes in this group had it not been for the secondary sepsis and multi-organ dysfunction. This highlights the importance of ongoing monitoring and prevention of health care associated infections which is a major cause of secondary worsening and mortality in many of these critical illnesses.

We did not find any difference in both groups with regard to key clinical outcomes such as duration of ventilation, inotropes, PICU stay, proportion with air leaks, proportion with shock, and use of adjuvant therapy or mortality. This could be attributed to the small numbers in our study. Our results suggest that using the PALICC criteria we may be able to identify more number of patients with ARDS early in the course of illness. Our findings are similar to the recently published study by Parvatheneni et al. (17) in which the outcomes did not differ between the “PALICC only” group and the “AECC or Berlin with or without PALICC” groups.

The strength of our study is that it adds to the literature on prevalence and outcome using the PALICC criteria in children. Even though data collection was retrospective, we included all patients with respiratory difficulty. However, there are several limitations to our study. Being retrospective in nature, there were missing data in few with difficulty in interpretation of pulse oximetry-based criteria. It was a single center study within a limited time frame; hence, results cannot be generalized especially to units without HFOV from resource restricted settings.

## Conclusion

In comparison to Berlin definition, the PALICC criteria identified more number of patients with ARDS. Proportion with severe ARDS and complications was greater in the “Berlin with or without PALICC” group as compared to the “PALICC only” group. There were no differences in clinical outcomes between the groups.

## Ethics Statement

This study was carried out in accordance with the recommendations of ICMR/GCP guidelines. We took waiver of consent as it was a retrospective study. The protocol was approved by the Institutional ethics committee, AIIMS, New Delhi, India.

## Author Contributions

Conception and design of work (SG, JS, RL, and SK); data acquisition (SG and JS); data analysis and manuscript revision and editing (JS); data interpretation (SG, JS, and RL); first draft of manuscript (SG); final approval of the version to be published, and agreement to be accountable for all aspects of the work (JS, RL, and SK).

## Conflict of Interest Statement

On behalf of all authors, the corresponding author states that there is no conflict of interest.
