# Supplementary material for: Comparison of Prevalence and Outcomes of Pediatric Acute Respiratory Distress Syndrome Using Pediatric Acute Lung Injury Consensus Conference Criteria and Berlin Definition
Source: Front Pediatr. 2018 Apr 9;6:93. doi: 10.3389/fped.2018.00093 (PMC5900438; doi:10.3389/fped.2018.00093)
Supplement: Supplementary file 1 [file table_1.DOCX]

**Electronic Supplement 1**

**Table. Clinical outcomes of patients of different levels of severity of ARDS by Berlin and PALICC criteria**

| **Variables** | **ARDS severity by P/F Ratio (‘Berlin with or without PALICC’ group) (n=26)** | | | **P value** | **ARDS severity by OI/OSI (‘PALICC only’ group) (n=32)** | | | **P value** |
| --- | --- | --- | --- | --- | --- | --- | --- | --- |
|  | **Mild (n=5)** | **Moderate (n=8)** | **Severe (n=13)** |  | **Mild (n=16)** | **Moderate (n=10)** | **Severe (n=6)** |  |
| **Duration of mechanical ventilation (hrs.) (Median, IQR)**   - Total duration - Duration of ventilation for ARDS | 8(8, 9)  7.5 (4.4, 13) | 4.8 (3.2, 16.5)  4.5 (2.3, 23) | 7.5 (3.5, 14)  6.8 (1.3, 10.3) | 0.82  0.92 | 9 (4.5, 13)  9 (4, 14) | 6 (3, 29)  4 (3, 7) | 2.4 (1.3, 7)  2.4 (1.3, 7) | 0.18  0.09 |
| **Air leaks (N, %)** | 1 (20) | 2 (25) | 4 (31) | 0.89 | 0 (0) | 2 (20) | 0 (0) | 0.09 |
| - Proportion of patients requiring HFOV (N, %) - Duration of HFOV (hrs) (Median, IQR) | 0 (0)  0 | 2 (25)  3.2 (0.4, 6) | 7 (58.3)  3 (0.4, 8) | 0.05  0.66 | 1 (6.3)  6.75 (2.5, 11) | 1 (10)  1.2 (0.4, 3.1) | 2 (33.3)  1.4 (0.3, 2.5) | 0.22  0.23 |
| **Need for dialysis (N, %)** | 1 (20) | 1 (12.5) | 3 (23) | 0.83 | 4 (25) | 3 (30) | 3 (50) | 0.58 |
| **Need for vasoactive therapy (N, %)** | 4 (80) | 8 (100) | 13 (100) | 0.11 | 12 (75) | 8 (80) | 6 (100) | 0.4 |
| - **Need for steroids (N, %)** - **Duration of steroids use (days) (Median, IQR)** | 3 (60)  14 (13, 48) | 6 (75)  3 (0.5, 4.5) | 12 (92)  3 (0.9, 8) | 0.26  0.07 | 9 (56.2)  4.75 (2, 7) | 5 (50)  9 (5, 9) | 6 (100)  2.3 (1, 3) | 0.1  0.1 |
| **Duration of ICU stay (days) (Median, IQR)** | 12.5 (9, 14) | 4.5 (3.5, 9.5) | 9.3 (3, 15) | 0.37 | 8.5 (6, 13) | 7 (4, 37) | 2 (1.2, 7) | 0.1 |
| **PELOD Score (Median, IQR)**   - Day 2 - Day 5 | 11 (2, 22)  6.5 (2, 11) | 11 (3, 12)  2 (1, 6.5) | 16 (10, 30)  11 (11, 20) | 0.51  0.15 | 16.5 (11, 22)  12 (0, 30) | 21 (12, 22)  11 (5.5, 27) | 30 (20, 30)  11 (11, 11) | 0.36  0.95 |
| - **Mortality (N, %)** - **Mortality due to ARDS (N, %)** | 1 (20)  0 (0) | 5 (62.5)  1 (20) | 9 (69)  6 (66.7) | 0.16  0.15 | 7 (43.75)  1 (14.3) | 5 (50)  1 (20) | 6 (100)  0 (0) | 0.05  0.54 |

Categorisation of severity of ARDS by OI/OSI Ratio

|  | Non Invasive ventilation (n=3) | Invasive ventilation (n=32) |
| --- | --- | --- |
| Mild |  | 16 |
| Moderate |  | 11 |
| Severe |  | 5 |
